# The complete plastid genome of an evergreen tree *Litsea elongata* (Lauraceae: Laureae)

**DOI:** 10.1080/23802359.2020.1778566

**Published:** 2020-06-17

**Authors:** Chao Liu, Huanhuan Chen, Lihong Han, Lizhou Tang

**Affiliations:** Key Laboratory of Yunnan Province Universities of the Diversity and Ecological Adaptive Evolution for Animals and Plants on Yungui Plateau, College of Biological Resource and Food Engineering, Qujing Normal University, Qujing, China

**Keywords:** *Litsea*, chloroplast, phylogeny

## Abstract

*Litsea elongata* (Nees) J. D. Hooker is an economically important timber and medicine tree. In this study, the complete plastid genome of *L. elongata* was assembled and analyzed. The plastid genome mapped a 154,027 bp circular DNA molecule with a GC content of 39.2%, consisting of a large single-copy region (LSC) of 93,688 bp, a small single-copy region (SSC) of 18,851 bp, and two inverted repeat regions (IRa and Irb) of 20,744 bp. A total of 127 genes were detected in the plastid genome, including eight ribosomal RNA (rRNA) genes, 36 transfer RNA (tRNA) genes, and 83 protein-coding genes. Phylogenomic analysis based on 39 complete plastomes of Laureae in the family Lauraceae supports the close relationships among *L. coreana*, *L. elongata*, *L. japonica*, and *L. pierrei*.

*Litsea elongata* (Nees) J. D. Hooker as an evergreen tree is naturally distributed in warmer regions of Asia in the family Lauraceae (http://foc.iplant.cn/). It belongs to the genus *Litsea* Lamarck which contains about 200 species distributed mainly in tropical or subtropical Asiaand North or South America (http://www.plantsoftheworldonline.org/). The relationships among the genera traditionally recognized within the monophyletic Laureae are still unclear, and *Laurus*, *Lindera*, and *Litsea* form several distinct and polyphyletic clades (Song et al. 2017; Liao et al. 2018; Zhao et al. 2018; Jo et al. 2019; Tian et al. 2019; Song et al. 2020). For a better understanding of the relationships of *L. elongata* and other Laureae species, we assembled and characterized the plastid genome of *L. elongata* as a resource for evolution and breeding research.

The leaf samples of *L. elongata* were collected from Hangzhou Botanical Garden (Zhejiang, China; Long. 120.118599 E, Lat. 30.253189 N, 31 m), which were used for DNA extraction (Doyle and Dickson 1987). The voucher was deposited at the Biodiversity Research Group of Xishuangbanna Tropical Botanical Garden (Accession Number: XTBG-BRG-SY36644). Genome was sequenced following Zhang et al. (2016), and their 15 universal primer pairs were used to perform long-range PCR for next-generation sequencing. The contigs were aligned using the publicly available plastid genome of *L. cubeba* (LAU00060) (Song et al. 2020). The plastid genome of *L. elongata* was assembled and annotated using Geneious 4.8 and GeSeq (https://chlorobox.mpimp-golm.mpg.de/geseq.html).

The whole plastid genome of *L. elongata* (LAU00121) was 154,027 bp in length The plastid genome comprised of a large single-copy (LSC) region (93,688 bp), a small single-copy (SSC) region (18,851 bp), and two inverted repeat (IRa and IRb) regions (20,744 bp). It contained 127 genes in the plastid, including 36 transfer RNA (tRNA) genes, 8 ribosomal RNA (rRNA) genes, and 83 protein-coding genes. The GC content of the whole plastid genome was 39.2%, and those of LSC region, SSC region, and IR region were 38.0%, 33.9%, and 44.3%, respectively.

Furthermore, the plastid genome sequences of *L. elongata* and other 38 species in Laureae were aligned by MAFFT v7.450. Maximum-likelihood (ML) phylogenetic analyses were performed base on GTR + F + R3 model in the iqtree version 1.6.7 program with 1000 bootstrap replicates (Figure 1). The ML phylogenetic tree with 52–100% bootstrap values at each node showed that *Litsea* species grouped into two clades, and that *L. coreana*, *L. cubeba*, *L. elongata*, *L. japonica*, *L. panamonja*, and *L. pierrei* were located in the same clade, while *L. glutinosa*, *L. magnifolia*, and *L. tsinlingensis* in another clade.

**Figure 1.**
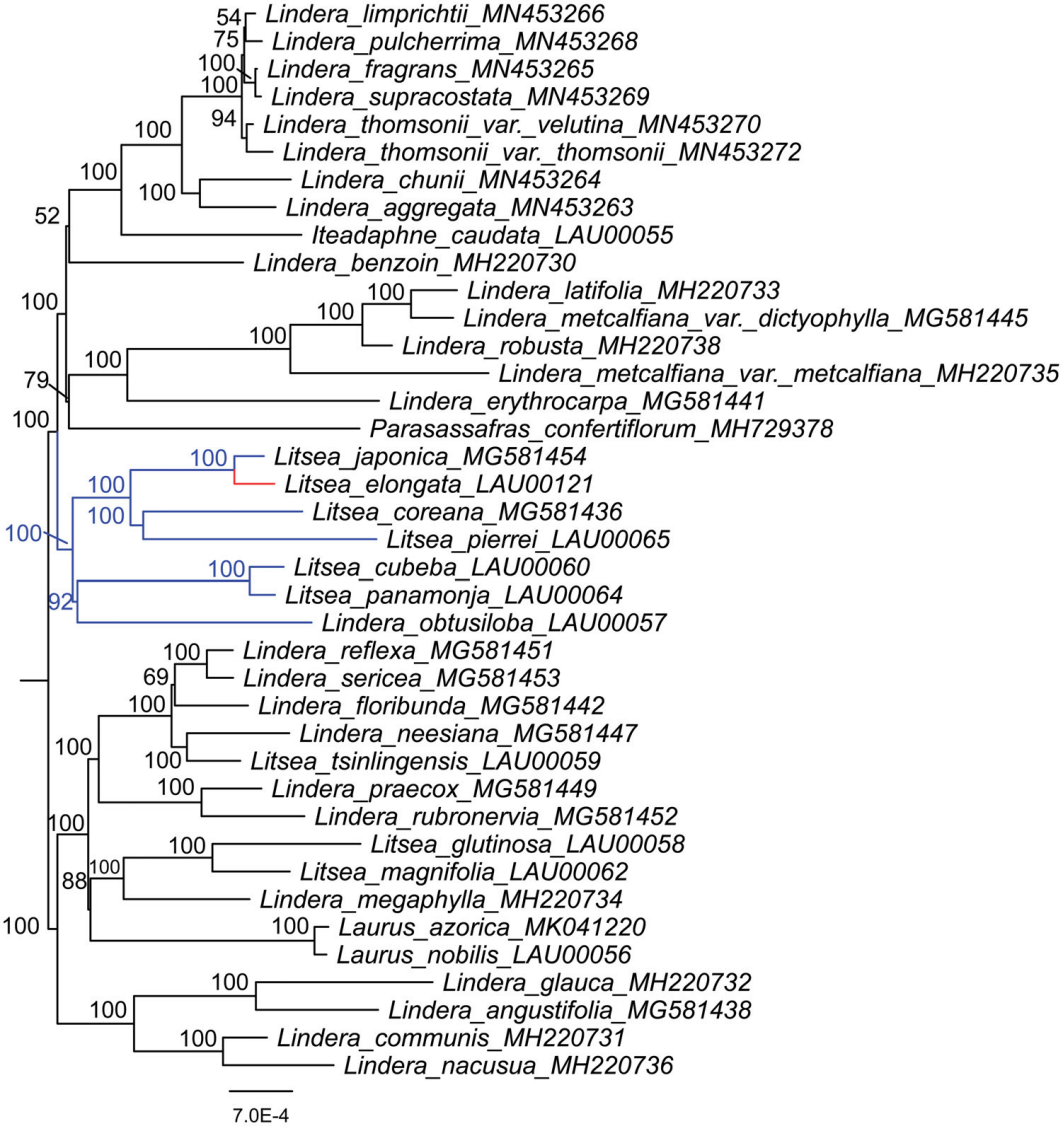
The maximum-likelihood phylogenetic tree constructed with plastid genomes of *L. elongata* and other 38 species.

## Data Availability

The data that support the finding of this study are openly available in Lauraceae Chloroplast Genome Database (http://lcgdb.wordpress.com). Accession number is LAU00121.
